# Is there a universal answering strategy for rejecting negative propositions? Typological evidence on the use of prosody and gesture

**DOI:** 10.3389/fpsyg.2015.00899

**Published:** 2015-07-07

**Authors:** Santiago González-Fuente, Susagna Tubau, M. Teresa Espinal, Pilar Prieto

**Affiliations:** ^1^Prosodic Studies Group, Department of Translation and Language Sciences, Universitat Pompeu FabraBarcelona, Spain; ^2^Department of English and German Philologies, Universitat Autònoma de BarcelonaBarcelona, Spain; ^3^Department of Catalan Philology, Universitat Autònoma de BarcelonaBarcelona, Spain; ^4^Institució Catalana de Recerca i Estudis AvançatsBarcelona, Spain

**Keywords:** truth-based answering systems, polarity-based answering systems, REJECT, prosody, gesture

## Abstract

Previous research has proposed that languages diverge with respect to how their speakers confirm and contradict negative questions. Taking into account the classification between truth-based and polarity-based languages, this paper is mainly concerned with the expression of REJECT (a semantic operation that signals a contradiction move with respect to the common ground, along Krifka's lines) in two languages belonging to two typologically distinct answering systems, namely Catalan (polarity-based) and Russian (a mixed system using polarity-based, truth-based, and echoic strategies). This investigation has two goals. First, to assess empirically the relevance of prosodic and gestural patterns in the interpretation of confirming and rejecting responses to negative polar questions. Second, to test the claim that in fact speakers resort to strikingly similar universal strategies at the time of expressing rejecting answers to discourse accessible negative assertions and negative polar questions, namely the use of linguistic units that encode REJECT in combination with ASSERT. The results of our investigation support the existence of a universal answering system for rejecting negative polar questions that integrates lexical and syntactic strategies with prosodic and gestural patterns, and instantiate the REJECT and ASSERT operators. We will also discuss the implications these results have for the truth-based vs. polarity-based taxonomy.

## Introduction

Unlike neutral questions, negative polar questions like *Is Jane not coming?* require non-neutral contexts, which means that they are produced when speakers have compelling evidence against some proposition (i.e., ￢*p*; Ladd, 1981; Büring and Gunlogson, 2000; Romero and Han, 2004; Reese, 2006). In this respect, negative questions have been traditionally described as biased questions, because the speaker in fact assumes ￢*p*.

It has been proposed in previous research that languages diverge with respect to how their speakers confirm and contradict negative questions (Kuno, 1973; Pope, 1976; Jones, 1999; Levinson, 2010; Holmberg, 2013; i.a.). As described by Jones (1999, pp. 9–11), the selection of different polarity particles to answer negative polar questions provides crucial evidence for two types of answering systems, which he calls *polarity-based systems* vs. *truth-based systems*. In polarity-based systems, such as the one exemplified for English in (1), both the responses to negative questions and the responses to positive questions are polarity-based in the sense that the positive or negative particles agree with the polarity of the elliptical (or non-elliptical) proposition of the answer (e.g., *No, I'm not* or *Yes, I am*)^1^. That is, if the answer particle is negative (1,A1), it will be expected to precede a full negative sentence, whereas if the answer particle is positive (1,A2), it will be expected to precede a full positive sentence. Since the particles *yes*/*no* may occur on their own, they are considered to be absolute polarity particles (Farkas and Bruce, 2010).

(1)  Q: *Aren't you staying?*
      A1. Confirming answer:         *No* [, I'm not].
      A2. Rejecting answer:            *Yes* [, I am].
      [examples from Jones (1999): 9, ex. (16)]

Conversely, in languages with a *truth-based system* (e.g., Chinese or Japanese; Jones, 1999: 8ff.) responses to negative questions, in contrast with responses to positive questions, are not polarity-based. Interestingly, these languages confirm the truth of the negative proposition by answering *yes* (i.e., the answer “agrees” with the whole content of the negative sentence), as in the confirming answer in (2,A1) and contradict the truth of the negative proposition by answering *no* (i.e., the answer “disagrees” with the whole content of the negative sentence), as in the rejecting answer in (2,A2).

(2)  Q: *Keoi-dei   m    jam  gaafe?* (Cantonese)
           he/she-PL not drink coffee?
          “Do they not drink coffee?”
      A1. Confirming answer: *Hai*.
                                          yes  “No [, they don't
                                                     drink coffee.]”
      A2. Rejecting answer: *M hai*.
                                       not yes “Yes [, they do.]”
      [examples from Holmberg (2013):33, exs. (6) and (9)]

The truth-based system has also been referred to as the *agreement/disagreement system*, which captures the fact that the speaker agrees to the negative proposition in the negative question when confirming with a *yes*-answer (e.g., *Yes, you are right, I do not drink coffee*), and disagrees to it when contradicting with a *no*-answer (e.g., *No, you are not right, I drink coffee*). In the earliest study on this topic, Pope (1976, p. 73) distinguished between a positive-negative answering system for languages like English, and an agreement-disagreement answering system for languages like Japanese (see also Kuno, 1973; Pope, 1976; Holmberg, 2013, p. 32).

There has been recent evidence that this bipartite typological classification is not so clear-cut. Research has shown that answering systems can also differ on a different linguistic dimension, e.g., on whether they are *echoic* (Portuguese) vs. *non-echoic* (English) (Jones, 1999), that is, on whether the responses to questions repeat the verb in the question or not. Slavic languages such as Russian and Czech are echoic languages. With respect to negative questions, the Russian echo-based response system behaves like a system which incorporates features of both truth-based and polarity-based systems. While the confirming answers in (3,A1) are polarity-based (the polarity of the response particle coincides with the polarity of the response), the rejecting answers in (3,A2) are echoic and truth-based, the negative particle reflecting a disagreement with the truth of the negative proposition in the question.

(3)  Q: V*y        ne vernete                        knigu?*
           you+pl no return+perfv+pres+2pl book
           “Will you not return the book?”
      A1. Confirming answer:   *Net*. /
                                            no
                                            *Net*, ne vernu.
                                            no   not return+perfv+pres+1sg
                                            “No [, I will not return it.]”
      A2. Rejecting answer: *Vernu*./
                                        return+perfv+pres+2pl
                                        *Net, vernu*.
                                        no    return+perfv+pres+2pl
                                        “Yes, [I will return it.]”
[examples from our Russian informants]

Catalan can initially be classified as a polarity-based language, since Catalan speakers confirm the truth of the negative proposition by answering *no* (i.e., the negative particle conveys the polarity of the answer and expresses “agreement” with the negative assumption in the question), as in (4,A1), and reject the truth of the negative proposition by answering *yes* (i.e., the positive particle expresses “disagreement” with the negative assumption in the question), as in (4,A2). The interpretation of a contradiction reading of a bare *sí* “yes” answer to a negative polar question can only be obtained if this particle is uttered with a “contradiction tune,” L+H^*^ L!H%. An alternative reply, purely echoic, is also illustrated in (4,A2).

(4)         Q: *No prenen              cafè?* (Catalan)
                  not drink.pres.3.pl coffee?
                 “They don't drink coffee (either)?”
             A1. Confirming answer: *No*.
                                                 not [“No, they don't.”]
             A2. Rejecting answer: - #*Sí*.
                                                yes [“Yes, they do.”]
                                                - *Sí*. _L+H^*^L!H%_
                                                yes [“Yes, they do.”]
                                                *En prenen*.
                                                Cl take [“Yes, they do.”]

Pilot production data collected for Catalan for the present investigation shows that speakers can also resort to a variety of strategies to contradict negative questions, some of them corresponding to non-polarity-based strategies. In essence, Catalan speakers can use a *no* response to a negative question not only to confirm the negation of the question (as in 5,A1), but also to reject it (as in 5,A2). Interestingly, an informal analysis of the data also reveals that the contradictory *no* answer in (5,A2) and the confirmatory *no* answer in (5,A1) are produced with distinct prosodic patterns. Even more interesting is the fact that Catalan positive particle *sí* “yes” can be used not only to confirm the negation of the question (as in 5,A3), but also to reject the negative presupposition of the negative question (as in 5,A4); similarly, both contradictory *yes* (5,A4) and confirmatory *yes* (5,A3) are produced with distinct prosodic patterns (see Section Prosodic Strategies for Catalan).

(5)          Q: *No  ha vingut, ta            mare?* (Catalan)
                   not has come,  your.sg.f mother?
                     “Has your mother not come yet?”
              A1: *No, no ha  vingut*.
                    not not has come.
                    “No, she hasn't come.”
              A2: *No, sí  que ha  vingut!*
                    not yes that has come!
                    “No, she has come!”
              A3: *Sí,  no  ha  vingut*.
                    yes not has come.
                    “Yes, she hasn't come.”
              A4: *Sí,  sí   que ha  vingut!*
                    yes yes that has come!
                    “Yes, she has come!”

Thus, it seems that in a polarity-based system such as Catalan *no* and *yes* answers can be used both to confirm the truth of a negative proposition (as in 5,A1 and 5,A3) and to deny or reject the truth of the same negative proposition (as in 5,A2 and 5,A4), and that these particles are produced with different prosodic patterns, depending on whether they are confirming or rejecting the presupposition associated to a negative question.

All in all, the abovementioned data show that the picture is more complex than the one depicted in (1) and (2) for the distinction between polarity-based and truth-based languages. In essence, the data show that polarity-based languages can use both *yes* and *no* not only to assert but also to contradict a previous assertion or assumption, indicating that the issue of the classification between truth-based and polarity-based languages deserves further attention. Moreover, it is clear that this classification has been primarily based on the use of lexical items, and that components like *prosody* and *gesture* have been largely ignored in the theoretical research. Yet, in the last decades, experimental researchers have convincingly shown that intonation and gesture patterns can imply different sets of pragmatic implicatures across languages (see Hirschberg, 2003; Wharton, 2009; Ebert et al., 2011; for a review). Intonation patterns have been shown to signal specific relationships between the speaker, the proposition uttered and the common ground, and to convey different epistemic commitments of discourse participants (e.g., Pierrehumbert and Hirschberg, 1990; Gunlogson, 2003; Beyssade and Marandin, 2006; Steedman, 2007; Portes et al., 2014; Krifka, in press). With respect to the encoding of reject or denial, recent work on Catalan has shown specifically how the pragmatic meaning of the contradiction tune (e.g., L+H^*^ L!H%) and also some specific types of gestures can be characterized as encoding presupposition denial of an activated negative discourse referent (e.g., Espinal and Prieto, 2011; Prieto et al., 2013; Tubau et al., 2015).

Overall these studies suggest that further research needs to be carried out for a full understanding of the various means that different languages have to express reject/denial/contradiction, and for a full understanding of the role of denial intonation and gesture patterns in polarity-based, truth-based, and echoic answering systems.

The general goal of this investigation will be to advance our understanding of the linguistic strategies used by speakers at the time of confirming and rejecting the input commitments of negative questions, with the general aim of understanding what the common strategies that speakers use are, and ultimately evaluating the validity of the postulated division between polarity-based and truth-based languages presented in this section. We are especially interested in investigating the potential role of denial and contradiction prosody and gestures as part of an underlying universal pattern of contradicting responses to negative questions. We chose to investigate Catalan and Russian, two languages with distinct answering systems: while Catalan exhibits mostly a polarity-based answering system (lexical particles combined with specific prosodic and gestural properties), Russian shows mainly an echoic answering system with a significant number of strategies characteristic of a truth-based system. In order to empirically investigate what the mechanisms used by native speakers of those languages are at the time of producing answers to negative questions, a Discourse Completion Task was run with 4 native speakers of Catalan and 4 native speakers of Russian. This methodology allowed us to obtain semi-spontaneous (but pragmatically controlled) natural responses to both positive and negative questions. The experimental results will help us gain insight on the linguistic strategies that languages use to confirm and contradict negative questions and, therefore, to shed some light on the boundaries of the typological difference initially established by Jones (1999) between polarity-based and truth-based systems. Moreover, the experimental results will help us test the claim that languages might use a common semantico-pragmatic strategy for rejecting negative propositions.

This article is organized as follows. Section Methods presents the methods and materials of our experiment. Section Results presents the results of the experiment. Finally, Section Discussion discusses our findings in relation to the typological distinctions mentioned above and also in relation to our main hypothesis on the common cross-linguistic semantic strategy in the answering systems to negative questions.

## Methods

A production experiment with native speakers of Catalan and Russian was conducted in order to obtain answers to positive and negative questions (and also natural verbal reactions to positive and negative assertions). Four speakers of each of these two languages participated in a Discourse Completion Task (henceforth DCT). This is a well-known procedure in the intercultural pragmatics literature which consists in using a situational prompt to elicit natural and contextualized responses from the participants (see Blum-Kulka et al., 1989; Félix-Brasdefer, 2010).

### Participants

Four native speakers of Central Catalan (4 women; mean age = 22.95; stdev = 4.22) and four native speakers of Russian (4 women; mean age = 30.75; stdev = 13.14) participated in a DCT. All Catalan participants were undergraduate students from the Universitat Pompeu Fabra in Barcelona. Catalan dominance was 70% (stdev = 7.07%) according to the participants' own reports of the estimate percentage of use of Catalan per day^2^. Russian participants were all from Moscow but recruited in Barcelona. According to their own reports, they had been living in Barcelona between 8 months and 4.5 years (mean = 1 year and 8 months), and they speak Russian on a daily basis with their families and friends (mean = 3.5 h/day).

### Materials

The main aim of the DCT production task was to obtain natural responses to positive and negative assertions and questions in Catalan and Russian, two languages that belong to two distinct typologies of answering systems, as described above.

For that purpose, we designed a DCT containing a set of 3 discourse contexts in different conditions. Each discourse context contained the combination of the two EXPERIMENTAL CONDITIONS, namely the POLARITY of the question or the assertion (POSITIVE vs. NEGATIVE), and the AGREEMENT of the answer with respect to the preceding proposition (CONFIRMING vs. REJECTING ANSWER). Table 1 illustrates one of the discourse contexts used for the DCT with the combination of the two conditions, namely (a) positive question/confirming answer, (b) positive question/rejecting answer, (c) negative question/confirming answer, and (d) negative question/rejecting answer. For the sake of presenting participants with a more varied set of DCT prompts, we repeated the same conditions using positive and negative assertions^3^.

**Table 1:** **Sample of one of the discourse contexts that served as a prompt for the DCT with the combination of the two conditions, e.g., a combination of POSITIVE/NEGATIVE QUESTIONS with corresponding CONFIRMING/REJECTING answers**.

**Situation:** Some time ago, your flatmate and you subscribed to a newspaper which is delivered every Saturday afternoon. Today is Saturday, but your flatmate will not be at home, so it is you who is responsible for opening the door to the deliveryman		
**Linguistic prompt**	**Polarity of the question**	**Agreement status of the answer**
(a) When your flatmate arrives at night, she asks you: **Has the deliveryman come?** You **confirm** that he has come. What would you say?	Positive question	Confirming answer
(b) When your flatmate arrives at night, the delivery man hasn't come yet. She asks you: **Has the deliveryman come?** You **deny** that he has come. What would you say?	Positive question	Rejecting answer
(c) When your flatmate arrives at night, she doesn't see the newspaper in the kitchen, where you usually leave it, and then she asks you: **Has the deliveryman not come yet?** You **confirm** that he hasn't come. What would you say?	Negative question	Confirming answer
(d) When your flatmate arrives at night, she doesn't see the newspaper in the kitchen, where you usually leave it. Actually, the deliveryman came, but you took the newspaper to your room and forgot to return it to the kitchen. As she doesn't see the newspaper in the kitchen, she asks you: **Has the deliveryman not come yet?** You **contradict** her. What would you say?	Negative question	Rejecting answer

Importantly, the target negative questions/statements under study only allow an inner negation interpretation. Thus, in the Catalan DCT contexts, while the target questions are compatible with negative polarity items such as *tampoc* “either,” they are not compatible with the positive polarity item *també* “also” (e.g., Catalan *No ha vingut el repartidor, tampoc?*, “Has the deliveryman not come, either?” vs. *#No ha vingut el repartidor, també?* “Has the deliveryman not come, too?” (see footnote 1)^4^. The target negative and positive questions and assertions were produced by two native speakers of each language and recorded using a PMD660 Marantz professional portable digital recorder and a Rode NTG2 condenser microphone in a quiet room at the Universitat Pompeu Fabra. This was done in order to ensure that all participants heard the imaginary interlocutor's question or assertion with the same acoustic properties and prosodic cues.

The materials in Catalan were translated into Russian with the help of our Russian informants. Crucially, the three discourse contexts used in the DCT were designed with a view to minimizing interferences of pragmatic variables across languages. Firstly, we chose common situations that most young people might be familiar with in their daily lives such as meeting with someone in a pub, meeting with friends to watch a football match, or having something delivered at home. Secondly, we made sure, by means of consulting native speakers of each language, that all the discourse contexts were perceived as normal in their respective cultures. Finally, we chose flatmates as interlocutors in the stories to make the imagined power relation between them and the informants as horizontal as possible.

Each participant thus received a complete set of 24 linguistic prompts [3 discourse contexts × 4 conditions × 2 prompt sentence types (i.e., question vs. assertion)]. See the Supplementary Material for a complete set of all the possible combinations (4 conditions × 2 prompt sentence types) of one of the three discourse contexts.

### Procedure

The DCT was conducted in a quiet room at the Universitat Pompeu Fabra. The 8 participants (4 Catalan speakers and 4 Russian speakers) were asked to stand in front of a Panasonic AG-HMC41 professional digital video camera, against a white background. Each of the participants was presented with a randomized presentation of the 24 stimuli containing all the possible combinations of experimental conditions and 6 fillers in two blocks, with a pause of 10 min between them.

The subjects first read a short set of instructions explaining the task and instructing them to reply as naturally as possible to the flatmate's target question and assertion with a short answer. For each item, participants began reading the target discourse contexts. When ready, they listened to the flatmate's utterance and immediately after they produced their answers. The whole session was video-recorded using a PMD660 Marantz professional portable digital player and later digitized at 25 frames per second, with a resolution of 720 × 576 pixels. The sound was sampled at 44,100 Hz using 16-bit quantization. Finally, either at the beginning or at the end of the experimental session, participants filled in a sociolinguistic questionnaire and signed a consent form.

A total of 240 responses were obtained for both languages. Ninety-six answers were obtained for the experimental conditions in each language (4 speakers × 3 discourse contexts × 4 conditions × 2 prompt sentence types), making a total of 192 experimental answers, plus 48 fillers (24 in each language).

### Measures and analyses

The collected data were entered on an Excel spreadsheet and submitted to lexico-syntactic, prosodic, and gestural analyses. The prosodic characteristics of the answers were analyzed with Praat (Boersma and Weenink, 2008) and coded following the Cat_ToBI system for Catalan (Prieto, 2014) and the ToRI system for Russian (Odé, 2003, 2008). With respect to gestures, ELAN was used for gesture annotation and aligned orthographic transcriptions of the video data. The guidelines in Allwood et al. (2005) and McNeill (1992) were used for coding gestural features.

## Results

This section presents the lexico-syntactic, prosodic, and gestural analysis of the confirming vs. rejecting answers obtained for positive and negative propositions in both Catalan and Russian.

### Catalan

#### Lexico-syntactic strategies

The target 96 Catalan responses were coded for lexico-syntactic strategies. The following types of lexico-syntactic strategies were identified:

1. An isolated *sí* “yes” answer.
2. An isolated *no* “no” answer.
3. *Sí* + explanation, in which we consider an explanation all the lexical material following the *sí* particle (e.g., *Ha vingut el repartidor? Sí, tens el diari per allà* “Has the deliveryman come? Yes, the newspaper is over there”).
4. *No* + explanation.
5. Repetition of *sí* “yes” (e.g., *sí, sí* “yes, yes” or *sí, sí, sí* “yes, yes, yes”).
6. Repetition of *no* “no.”
7. A reinforced positive answer such as *Clar que sí* “yes, of course.”
8. A reinforced negative answer such as *Que va, encara no!* “You must be joking, not yet.”
9. *Sí que* “yes that” + positive sentence.
10. The structure *No, sí que* + positive sentence', in which the verb uttered in the positive sentence is the same verb contained in the question (e.g., *No ha vingut, el repartidor? No, sí que ha vingut* “Has the deliveryman not come? No, he has come”).
11. *No* + negative sentence (e.g., *No, encara no ha començat* “No, it hasn't begun yet”).

Finally, Catalan speakers sometimes used a combination of some of the strategies reported above (e.g., *No, sí, sí que ha vingut* “No, yes, yes she has come,” which can be analyzed as the combination of the strategy in (10) “*No, sí que* + positive sentence” and in (5) repetition of *sí* “yes”).

As expected for a polarity-based language, rejecting answers to positive propositions and confirming answers to negative propositions were mostly produced with *no* “no” answers. Thus, answers with an explicit *no* “no” (e.g., isolated *no* answers, and *no* followed by an explanation or by a negative sentence) conform 79.2% of the rejecting answers to positive propositions and 87.5% of the confirming answers to negative propositions. By contrast, confirming answers to positive propositions and rejecting answers to negative propositions were mostly produced with *sí* “yes” and *sí* “yes” followed by an explanation (which conform 86% of the confirming responses and 46% of the rejecting responses to negative questions). It is immediately clear from these percentages that the strategies used to reject negative propositions are more varied than the strategies used in the other three conditions and, specifically, that the *sí* “yes” particles used differ from the *sí* “yes” particles used as simple confirming conditions to positive propositions [for example, Catalan speakers employed an isolated yes-answer more often when confirming a positive proposition (54.2%) than when rejecting a negative proposition (29.2%)]. The results of a chi-square test showed a near-significant trend (*p* = 0.07) for the presence of isolated *sí* “yes” responses in rejecting vs. confirming answers [χ^2^(1, 48) = 3.08, *p* = 0.07].

**Figure 1:**
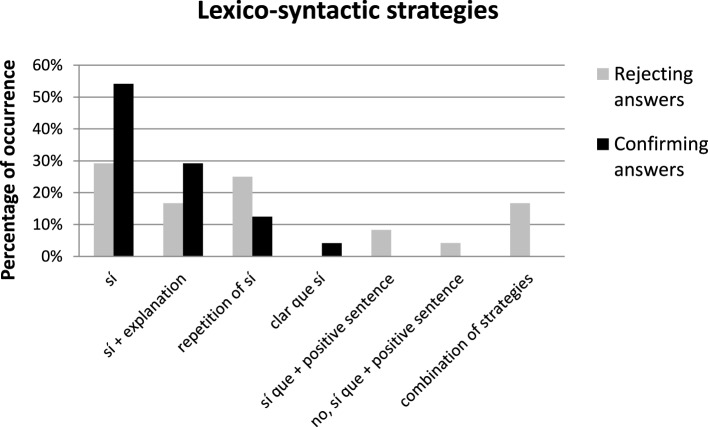
**Percentage of occurrence (y-axis) and type of lexico-syntactic strategies (x-axis) used by Catalan speakers to confirm positive questions/assertions (black columns) and to reject negative questions/assertions (gray columns)**.

Given this, we would like to now focus our attention on how Catalan speakers reject negative propositions and how these strategies differ from the *sí* strategies on the types of strategies used to confirm positive propositions. Figure 1 focuses on this comparison by showing the lexico-syntactic strategies used by Catalan speakers to reject negative questions/assertions (gray columns) and to confirm positive questions/assertions (black columns), as well as their frequency of occurrence.

At the time of confirming, Catalan speakers use fewer strategies than at the time of rejecting. When rejecting negative propositions, Catalan speakers most frequently use a *yes*-answer (e.g., *No ha vingut, el repartidor? Sí, ja ha vingut* “Hasn”t the deliveryman come? Yes, he has'). In the rejecting condition, participants employed a variety of lexico-syntactic strategies, as well as a combination of some of these strategies. While an isolated *sí* “yes,” the repetition of *sí* “yes” and the production of *sí* “yes” followed by an explanation were the most widely used strategies (with 46% of the data), other strategies were unique in this condition. These are, ordered according to the frequency with which they occur, a combination of strategies (16, 7%); “*Sí que* +positive answer” (“yes that + positive answer”) (8.3%); and “*No, sí que* + verb” (“No, yes that + verb”) (4.1%). It is interesting to note that *no*-answers, which have been reported to be general in truth-based languages, are also documented in some cases (in 8.2% -and in one case combined with another strategy-) and always immediately preceding a *sí que* “yes that” expression. This situation contrasts with the confirming responses to positive propositions, which are overwhelmingly answered through the use of *sí* “yes” (e.g., *Ja ha vingut, el repartidor? Sí, ja ha vingut* “Has the deliveryman come? Yes, he has”). The most widely used strategy to confirm positive presuppositions is an isolated *yes*-answer *sí* “yes” (54.2%). The second most popular strategy is the use of a *yes*-answer followed by an explanatory comment (29.2%). With much lower percentages we observe the repetition of a *yes*-answer *sí, sí* (12.5%), and the use of reinforced confirming particles of the sort *clar que sí* lit. “of course that yes” (4.1%).

In the next section we investigate the prosodic and gestural strategies that Catalan speakers used in confirming vs. rejecting answers, given what we know from the intonation (and gestural) patterns of *yes*-answers (see Tubau et al., 2015).

#### Prosodic strategies

This subsection is aimed at assessing the intonational strategies Catalan speakers employed for contradicting discourse assumptions to previous negative propositions and compare them with the prosodic patterns used in confirming answers to positive propositions. To do this, our prosodic analysis focused on the intonational patterns which were associated with the polarity particle *sí* “yes” appearing either in isolated *yes*-answers or in *yes*-answers produced in a single intonational phrase (e.g., separated by a pause from the following intonational phrase). A total of 87.5% of confirming answers to positive propositions and 54.2% of rejecting answers to negative propositions met this criterion.

**Figure 2:**
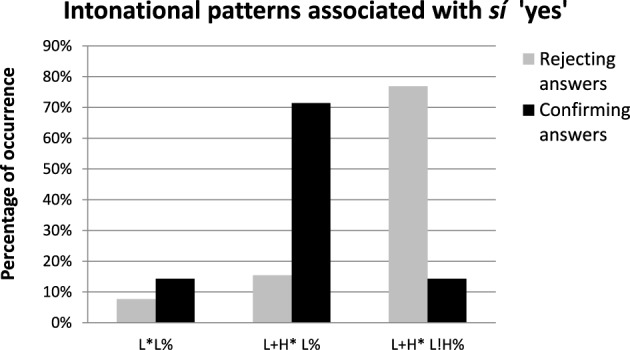
**Percentage of occurrence of intonational patterns associated with** ***sí*** **“yes” used by Catalan speakers to confirm positive propositions (black columns) and to reject negative propositions (gray columns)**. The data correspond to answers that are produced in a single intonational phrase.

Figure 2 shows the intonational patterns associated with *sí* “yes” as used by Catalan speakers to confirm positive propositions (black columns) and to reject negative propositions (gray columns), as well as their frequency of occurrence. Three types of intonational patterns were found in the data, namely L^*^ (falling tune), L+H^*^ L% (rising-falling tune), and L+H^*^ L!H% (a rising-falling-rising tune). While the first two have been typically associated with broad-focus assertions, the latter has been called the “contradiction tune” (Espinal and Prieto, 2011).

The results in Figure 2 illustrate an important distinction between the intonational strategies employed to confirm a positive proposition vs. to contradict a negative one. Thus, whereas Catalan speakers confirmed a positive proposition by using mainly broad-focus statement intonation (71% of L+H^*^ L% and 14% of L^*^ L% intonation patterns), they mainly used the L+H^*^ L!H% pattern when they had to contradict a negative presupposition (in 77% of cases). The results of two chi-square tests showed that the presence of L+H^*^ L% and L+H^*^ L!H intonational patterns was significantly related to the type of answer in which these patterns were produced (confirming vs. rejecting) [χ^2^(1, 34) = 10.08, *p* < 0.01 for L+H^*^ L% pattern; and χ^2^(1, 34) = 13.33, *p* < 0.01 for L+H^*^ L!H].

Thus, results show that when Catalan speakers reject a negative presupposition by uttering a *yes*-word with a single intonational phrase, they mainly employ the L+H^*^ L!H% contour. This L+H^*^ L!H% nuclear configuration pattern has been found in previous studies (Espinal and Prieto, 2011; Tubau et al., 2015) to act as a relevant prosodic marker used in the contradiction of negative presuppositions associated with negative questions.

#### Gestural strategies

**Figure 3:**
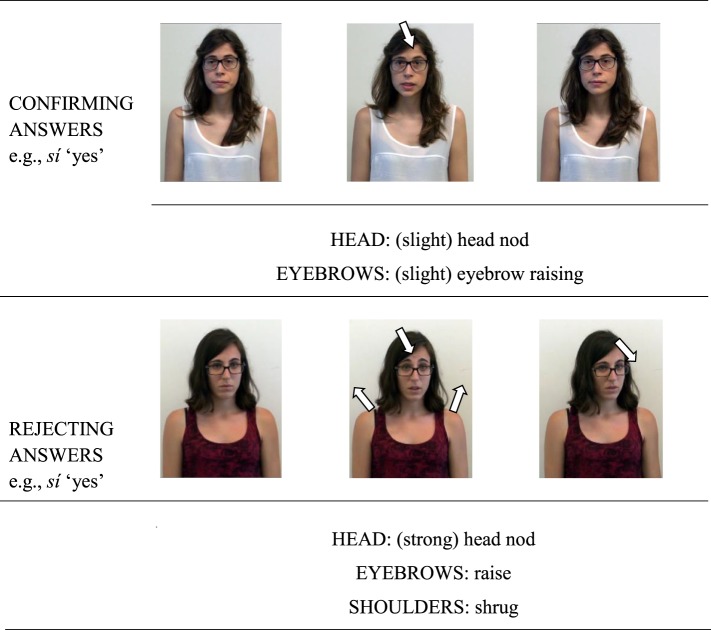
**Two image sequences of the typical gestures associated with Catalan** ***sí*** **“yes” in confirming answers to positive propositions (top panel) and in rejecting answers to negative propositions (bottom panel)**.

This subsection presents the analysis of the gestural patterns produced together with the *yes*-particles in rejecting and confirming answers. As in the case of prosodic analyses, only gestures performed together with isolated *yes*-answers and *yes*-answers produced in a single intonational phrase (e.g., separated by a pause from the following intonational phrase) were analyzed. The coded gestures involved head movements (e.g., head nod, head tilt, head shake), eyebrow movements (eyebrow raising), shoulder movements (e.g., shoulder shrug), as well as degrees of emphasis of some of the target movements (slight vs. strong head nod). Figure 3 shows two image sequences of the typical gestures associated with Catalan *sí* “yes” in confirming answers to positive propositions (top panel) and in rejecting answers to negative propositions (bottom panel).

**Figure 4:**
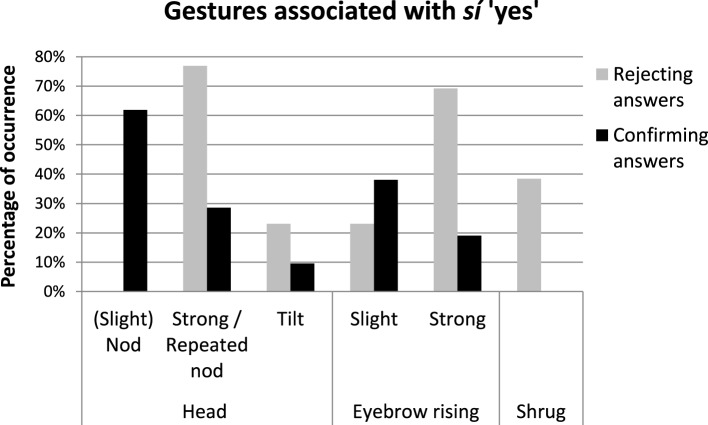
**Percentage of occurrence (y-axis) of the types of gestures (x-axis) used by Catalan speakers to confirm positive propositions (black columns) and to reject negative propositions (gray columns)**. The data correspond to gestures associated with *sí* “yes” that are produced in a single intonational phrase.

Figure 4 shows the percentage of occurrence of different gesture types used by Catalan speakers to confirm positive propositions (black columns) and to reject negative propositions (gray columns). The results show that most of the confirmation answers to positive questions/assertions were performed with some type of head nod (e.g., 62% of the cases with slight head nods, and 29% of the cases with strong or repeated head nods) and with 10% of head tilts. Also, some *yes* particles were accompanied with slight (38%) or strong eyebrow raising (19%), whereas 43% present no eyebrow movements. By contrast, gestural marks produced together with *yes* particles in rejecting answers were associated with more marked head movements such as repeated or strong nods (77% of the cases) or head tilts (23.2%), and with a more consistent presence of eyebrow raising (92% of the cases –with 23% of slight and 69% of strong eyebrow raising-), as well as with shoulder shrugging (38.5%)^5^. The results of four chi-square tests showed that the presence of slight nods [χ^2^(1, 34) = 6.87, *p* < 0.01], strong/repeated nods [χ^2^(1, 34) = 7.53, *p* < 0.01], strong eyebrow raising [χ^2^(1, 34) = 8.56, *p* < 0.01] and shrug [χ^2^(1, 34) = 9.46, *p* < 0.01] were significantly related to the type of answer in which they were produced (rejecting vs. confirming).

In sum, the data in this section have shown how Catalan speakers display distinct lexico-syntactic strategies to confirm and to reject positive and negative propositions. Crucially, a different set of prosodic and gestural strategies were documented for the confirmation and rejection operators too.

### Russian

#### Lexico-syntactic strategies

The target 96 Russian responses were coded for lexico-syntactic strategies. The following types of lexico-syntactic strategies were identified:

1. An isolated *da* “yes” answer.
2. An isolated *net* “no” answer.
3. A reinforced *net* “no” answer (e.g., *Uzhe net*, “not yet”).
4. *Da* “yes” + explanation', in which we consider an explanation all the linguistic material following the *da*-word whereby speakers add some information about the context (e.g., *Maria prishla? Da, ona zdes* “Has Mary arrived? Yes, she is here”).
5. *Da* “yes” + echoic verb (e.g., *Kurier prikhodil? Da, on prikhodil* “Has the deliveryman come? Yes, he has come”).
6. Repetition of *da* “yes” + echoic verb (e.g., *Da, da, ona prikhodil* “yes, yes, she has arrived”).
7. Echoic verb (i.e., the same verb uttered in the question).
8. Repetition of the echoic verb.
9. *Net* “no” + echoic verb (e.g., *Net, prikhodil* lit. “No, has come”).
10. *Net* “no” + explanation (e.g., *Maria uzhe ne prishla? Net, ona zdes* “Has Mary not arrived yet? No, she is here”).
11. Repetition of *net* “no” + explanation.
12. *Net* “no” + negative sentence, in which the verb uttered in the negative sentence is the same verb contained in the question (e.g., *Kurier uzhe ne prikhodil? Net, on ne prikhodil* “Has the deliveryman not come yet? No, he has not come”).
13. Echoic verb + explanation (e.g., *Uzhe nachalsia, no est problemy s'televizorom* “It has started, but there are some problems with the television”).
14. Positive sentence, in which the verb uttered in the positive sentence is the same verb contained in the question.
15. “Other,” in which the sentence uttered does not explicitly confirm or contradict the linguistic prompt (e.g., *Oi, zabyla vernut* “Oh, I've forgotten it”).

Given the mixed status of Russian, we expected a different pattern of results from Catalan. First, as in Catalan, rejecting answers to positive propositions were mostly produced with an isolated or reinforced *net* “no” answer or a *net* “no” followed by a negative sentence (these three strategies conform 70% of the responses in this condition). A similar pattern of results is found for confirming answers to negative propositions, where speakers mainly used the *net* “no,” and *net* “no” followed by a negative sentence or by an explanation (80% of the cases). By contrast, confirming answers to positive questions were mostly produced with *da* “yes,” *da* “yes” followed by an explanation or by an echoic verb (80% of the cases). Now, focusing on the types of strategies used to reject negative assumptions of negative polar questions, and in contrast with the Catalan case, they were never produced with a *da* “yes” particle. Most of the responses (48%) were produced with a *net* “no” followed by an echoic verb structure or just with the isolated echoic verb. This is the expected pattern in truth-based languages, where the *net* “no” particle encodes a disagreement or rejection of the negative proposition that is the most salient one in the common ground.

**Figure 5:**
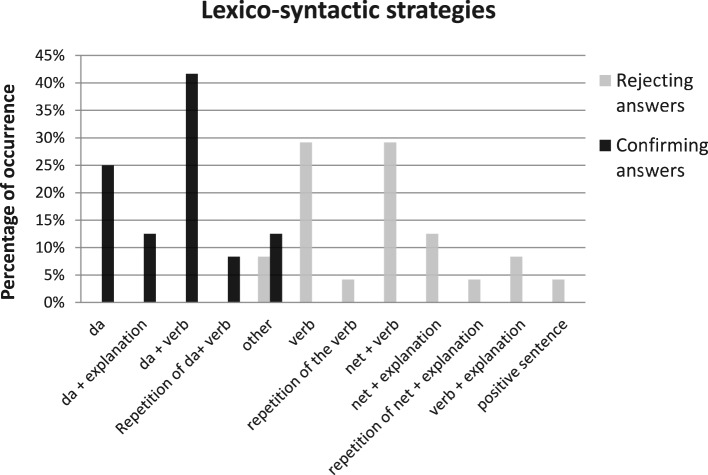
**Percentage of occurrence (y-axis) and type of lexico-syntactic strategies (x-axis) used by Russian speakers to confirm positive propositions (black columns) and to reject negative propositions (gray columns)**.

Let us now focus on the Russian rejection strategies to negative propositions. Figure 5 compares the lexico-syntactic strategies used by Russian speakers to reject negative propositions (gray columns) with the strategies used to confirm positive propositions (black columns), as well as their frequency of occurrence.

As mentioned before, the strategies that Russian speakers employed for contradicting negative assertions/questions (a) were different from confirming answers to positive propositions, where the word *da* “yes” is mostly used; and (b) were very different from those employed by Catalan speakers: whereas Catalan speakers mostly used expressions containing the word *sí* “yes” (95.8%), Russian speakers mainly used expressions containing the particle *net* “no” (45.9%) or a sentence beginning with an echoic verb (41.7%). Russian participants did not use the word *da* “yes” in any of the responses produced to react against a negative proposition asserted in the discourse context or assumed from it. Here we list the particular strategies used for rejecting negative propositions, ordered according to the frequency in which they occurred: *net* “no” + echoic verb (29.2%), echoic verb (29.2%), *net* “no” + explanation (12.5%), echoic verb + explanation (e.g., *Uzhe nachalsia, no est problemy s'televizorom* “It has started, but there are some problems with the television”) (8.3%), other strategies (8.3%), repetition of the verb (4.2%), repetition of *net* “no” + echoic verb (4.2%), and a positive sentence (4.2%).

#### Prosodic strategies

This subsection has the aim of assessing the intonational strategies Russian speakers use to reject negative propositions and compare them to the patterns used to confirm positive propositions. To do this, our prosodic analysis focused on the intonational patterns which were associated with the word *net* “no” or with an echoic verb in the rejecting condition, and with the word *da* “yes” in the confirming condition. This strategy allows us to control for potential structural effects on intonation. As in the case of Catalan, all target words had to form a single intonational phrase (i.e., the words had to be found in isolation or separated by a pause from the following intonational phrase). The abovementioned patterns produced in an intonational phrase constitute in fact the majority of the Russian responses (e.g., a total of 66.6% of confirming answers to positive propositions and 62.5% for rejecting answers to negative propositions met this criterion).

**Figure 6:**
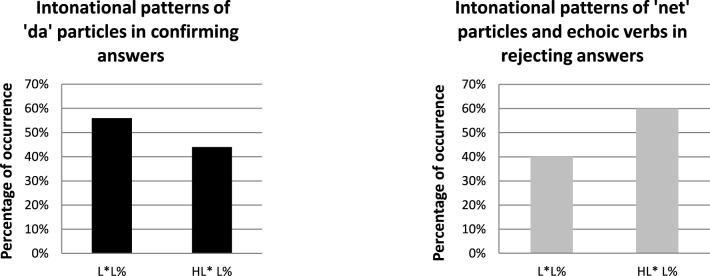
**Percentage of occurrence of intonational patterns associated with** ***da*** **“yes” particles used by Russian speakers to confirm positive propositions in confirming answers (black columns—left panel), and to intonational patterns associated with** ***net*** **“no” particles or echoic verbs used to reject negative propositions (gray columns—right panel)**. As we did for Catalan, we analyzed all the data containing particles or echoic verbs produced in a single intonational phrase and independently from the lexical strategy in which they appear.

Figure 6 shows the frequency of occurrence of the intonational patterns associated with the word *da* “yes” in the confirmation condition and with *net* “no” or the echoic verb in the rejecting condition. Two types of intonational patterns were found in the data. According to Odé (2008), L^*^ is a steep fall from a high or a mid-level tone, and its main communicative function is to express completeness or neutral finality. The pitch accent HL^*^ is realized as a steep fall beginning at the onset of the accented vowel and its main communicative function is to express completeness with emphasis.

In general, the prosodic results for the Russian speakers show that even though HL^*^ L% is more frequently used in the rejecting condition (60%) than in the confirming condition (44%) [and conversely, that L^*^ L% is more often used for confirmation (56%) than for rejection (40%)], both patterns are consistently used both to confirm positive questions/assertions and to reject negative ones. The results of a chi-square test showed no significant differences between the presence of L^*^ L% or HL^*^ L% in rejecting or confirming answers [χ^2^(1, 31) = 0.81, *p* = 0.36]. Thus, we can conclude that despite the tendency for more emphatic HL^*^ L% patterns to be associated with rejections, the two intonational patterns are used in both the rejection and confirmation conditions.

#### Gestural strategies

**Figure 7:**
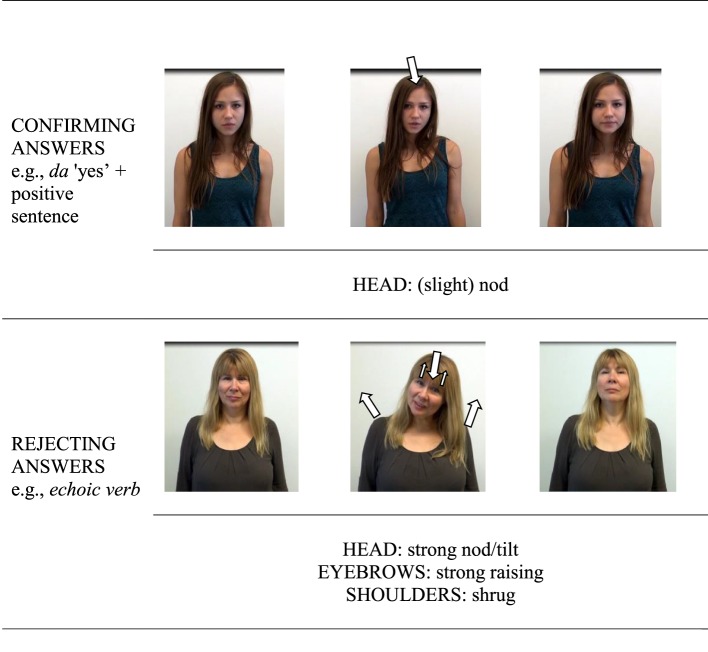
**Two image sequences of the typical gestures associated with Russian** ***da*** **“yes” confirming answers to positive propositions (top panel) and rejecting answers to negative propositions (bottom panel)**.

This subsection presents the analysis of the gestural patterns produced together with the Russian *da* “yes”-particles in the case of confirming answers and gestures produced together with the negative particle *net* “no” or with the echoic verb in the case of rejecting answers. As in the case of prosodic analyses, only gestures performed together with isolated *yes*- or *no*-answers and *yes*-answers produced in a single intonational phrase (e.g., separated by a pause from the following intonational phrase) were included in the analysis. As in the case of Catalan, coded gestures involved head movements (e.g., head nod, head tilt, head shake), eyebrow movements (eyebrow raising), shoulder movements (e.g., shoulder shrug), as well as degrees of emphasis of some of the target movements (slight vs. strong head nod). Figure 7 shows two image sequences of the typical gestures associated with Russian *da* “yes”/*no* “no” and echoic verbs in confirming answers to positive propositions (top panel) and rejecting answers to negative propositions (bottom panel).

**Figure 8:**
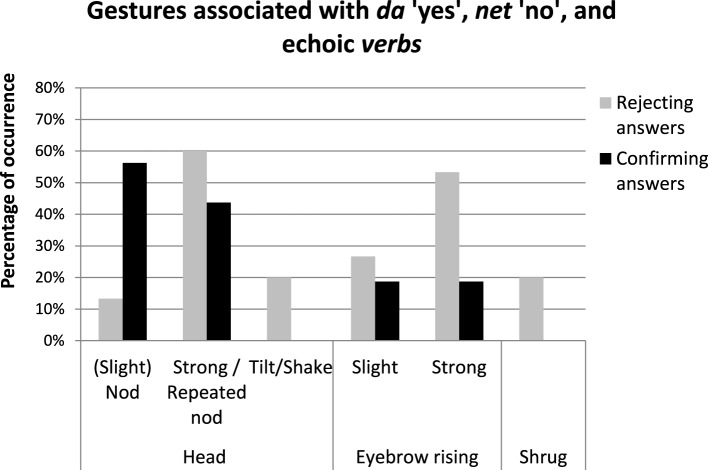
**Percentage of occurrence (y-axis) of the types of gestures (x-axis) used by Russian speakers to confirm positive propositions (black columns) and to reject negative propositions (gray columns)**. The data correspond to gestures associated with *da* “yes,” *net* “no” and echoic verbs that are produced in a single intonational phrase.

Results show that Russian speakers, like Catalan speakers, used head nod movements to confirm positive propositions (i.e., slight nods in 56% of the cases, strong/repeated nods in 44% of the cases), as well as eyebrow raising (38% of the cases, separated into slight movements in 19% of the cases and strong movements in 19% of cases). As for rejecting answers, gestural marks produced together with *no* and *verb* particles were associated with more marked head movements such as repeated or strong nods (60% of the cases) or head tilts (20%), and with a more consistent presence of eyebrow raising (in 80% of cases, separated into 27% of slight and 53% of strong eyebrow raising), as well as with shoulder shrugging (20%) (See Figure 8). The results of four chi-square tests showed that the presence of slight nods [χ^2^(1, 31) = 6.22, *p* < 0.01] and strong eyebrow raising [χ^2^(1, 31) = 4.04, *p* < 0.05] was significantly related to the type of answering conditions in which they were produced (rejecting vs. confirming). Similarly, the presence of tilt/shake and shrug were both approaching an acceptable significance level [tilt/shake χ^2^(1, 31) = 3.54, *p* = 0.059 and shrug χ^2^(1, 31) = 3.54, *p* = 0.059].

The data in this section have shown how Russian speakers display a distinct set of lexico-syntactic and prosodic strategies for the encoding of confirming and rejecting answers. Interestingly, while the two languages under study (that is, Catalan and Russian) differ in the lexico-syntactic and prosodic strategies used, gestural strategies were common in the two languages.

## Discussion

### Discussion of the data

The results of our investigation have revealed that Catalan and Russian show distinct types of answering systems if we take into account exclusively lexico-syntactic and prosodic strategies, but they use common gestural strategies. In the expression of reject whereas Catalan speakers mainly used the word *sí* “yes” or structures involving *sí* “yes” followed by an explanation or the repetition of the *sí* “yes” (95.8%), Russian speakers preferred to use expressions containing the particle *net* “no” –e.g., either *net* “no” followed by an echoic verb or an explanation (45.9%) or the standalone echoic verb (41.7%). This behavior constitutes the main grammatical difference between the two languages. However, even though Catalan has been shown to be a basic polarity-based language, it displays a mixed answering system for contradicting negative questions, since native speakers use both a *sí* “yes” answer with a specific tune (a polarity-based strategy) and less commonly a *no, sí* “no yes” answer (a truth-based strategy) to reject negative propositions. Similarly, Russian also shows a mixed answering system whereby rejections to negative propositions can be commonly produced by means of an echoic verb alone, or by *net* “no” in combination with an echoic verb.

Interestingly, the analysis of gestural patterns has shown how common denial gestures associated with the notion of reject are in both languages. Both Catalan and Russian speakers typically produce rejections to negative propositions with strong or repeated nods (78% of cases in Catalan and 60% of the cases in Russian). Also in both languages gestural marks accompanying rejecting answers are associated with a more consistent presence of eyebrow raising (92% in rejecting vs. 57% in confirming answers in Catalan; 79 vs. 42% in Russian), as well as shoulder shrugging (38.5 vs. 0% in Catalan and 20 vs. 0% in Russian) or shake/tilt head gestures (23 vs. 10% in Catalan and 20 vs. 0% in Russian). The abovementioned results suggest that the classical taxonomies proposed for answering systems provide little support at the time of understanding how these answering systems work across languages. Accordingly, we pursue the hypothesis that a full comprehension of the answering systems encountered in natural languages can only be achieved if grammatical (i.e., lexical and syntactic) strategies are analyzed together with intonational and gestural patterns.

In the following section we provide our semantico-pragmatic analysis of the data.

### Analysis

Our analysis of the data will be framed within a *theory of speech act dynamics*^6^. As proposed by Krifka (2013) (who follows Hamblin, 1971; Stalnaker, 1978; Gazdar, 1981; Alston, 2000; Gunlogson, 2003; Harnish, 2005; Beyssade and Marandin, 2006; among others), speech acts create spaces of commitments, and by means of them interlocutors may also introduce changes of commitments, in a dynamic and dialogical way^7^.

In everyday conversation, by uttering a linguistic expression, speakers are committed to various sorts of speech acts: assertions, questions, requests, warnings, etc. In our study we are specifically interested in four types of speech acts: requests, assertions, confirmations and rejects, which will be referred to by means of the operators REQUEST, ASSERT, CONFIRM, and REJECT. A REQUEST expressed by a speaker via a negative polar question is an enquiry about ￢*p*. An ASSERT speech act is claimed to express two commitments (Krifka, 2013, in press): one by which the speaker first expresses a commitment to the proposition (S_1_: *p*), and a second one by which the speaker calls on the addressee to be also committed to the same proposition, with the result that *p* becomes part of the common ground (*p* ∈ CG). A CONFIRM speech act is such that the speaker expresses the *same* commitment already expressed by the ASSERT speech act (cf. Farkas and Bruce, 2010). Finally, a REJECT speech act is one by which a speaker opposes to the commitment suggested by the interlocutor, and forces a change of commitment. Crucially, both CONFIRM and REJECT speech acts apply to an ASSERT speech act, in which the polarity of the sentence is expressed. Consider (6), which introduces a request with respect to a negative proposition followed by four possible answers. Both (6Q) and (6,A1) to (6,A4) are from Krifka (2013, ex. 16). On the right hand side, we represent the same negative polar question and the four possible answers in terms of the postulated operators.

**Table:** 

(6)	Q: Did Ede not steal the cookies?	[REQUEST did [ASSERT [Ede not steal the cookies]]]
	A1. Yes[SAME], he didn't.	[CONFIRM yes [ASSERT [he didn't]]]
	A2. Yes[+], he did.	[ASSERT yes [he did]]
	A3. No[REVERSE], he did.	[REJECT no [ASSERT [he did]]]
	A4. No[-], he didn't.	[ASSERT no [he didn't]]

Notice that the *yes* particle expresses either CONFIRM or ASSERT (positive polarity), and the *no* particle expresses either REJECT (i.e., reverse; cf. Farkas and Bruce, 2010; Roelofsen and Farkas, To appear) or ASSERT (negative polarity). REQUESTS, CONFIRMS, and REJECTS are to be considered as meta speech acts. By means of a REQUEST operator the speaker (S1) asks the addressee (S2) to perform a certain speech act. The operators CONFIRM and REJECT, in turn, apply to an ASSERT speech act that contains either *p* or ￢*p*.

We assume that participants in a conversation should be able to adequately infer whether their speech acts are being accepted or rejected by their interlocutors. Both assertions and questions (either positive or negative) can, therefore, be seen as functions that connect different commitments in a conversation (Krifka, in press). In the production experiments that we ran, assertions and questions are to be regarded as functions that connect to output commitments expressing either a confirming or a rejecting state. Furthermore, participants in normal conversations address two levels of semantic meaning, namely, (a) whether the answer is positive or negative, and (b) whether the response confirms or rejects the interlocutor's proposal. The former corresponds to the task of the ASSERT operator. The latter corresponds either to the confirmation of a propositional discourse referent that was introduced or assumed by the interlocutor, or to the rejection of a negative assumption.

With this framework in mind, we initially hypothesized the presence of a universal pragmatic optimization strategy by which confirming answers to negative questions tend to be optimally composed of a CONFIRM plus an ASSERT operator, and rejecting answers are optimally composed of a REJECT plus an ASSERT operator.

The data obtained in our experiments show that speakers of both languages follow a general pragmatic communicative strategy by which they REQUEST on an ASSERTed negative proposition, and interlocutors react against a previous negative proposition by combining these two speech act operators: REJECT and ASSERT. Our results show that two distinct languages share some universal rejecting answering strategies, namely the use of speech act particles in combination with full sentences and the combination with specific gestures (e.g., shrugging, eyebrow rising, head nodding). Still, they differ in other strategies: the presence of marked intonation patterns (e.g., L+H^*^ L!H% in Catalan), and the presence of an echoic strategy (in Russian). Overall, a clear pressure arises in these two languages to grammatically mark the reject or denial component of the answer. Thus, in the case of Catalan, the rejecting component is mostly instantiated through the tune pattern L+H^*^ L!H%, in optional combination with specific gestural patterns (i.e., strong and/or repeated head nods, eyebrow raising, and shrugging). In the case of Russian, the negative particle *net* is very often used sentence-initially to encode disagreement, in combination with gestural patterns that also encode a contradiction meaning (i.e., shakes, tilts, strong nods, and shrugs). In what follows we would like to propose a semantico-pragmatic analysis of the underlying pattern of contradicting responses to negative propositions that we obtained, which includes a compositional analysis of rejection and assertion speech acts uttered by speakers in the course of a conversation.

#### Catalan

Let us consider first the following negative question in Catalan:

(7) S1 to S2: *No ha vingut?* “Has he/she not come?”
    [_ForceP_REQUEST_S1,S2_ [_ForceP_ ASSERT_S1,S2_ [_NegP_ no [_TP_ ha vingut]]
    This negative question introduces two potential propositional discourse referents:
    φ = “he/she has come,” corresponding to TP
    ψ = ￢“he/she has come,” corresponding to NegP

However, (7) being a negative question biased toward a negative proposition (i.e., the speaker S1 at the time of expressing the negative interrogative sentence assumes *p*), the meaning of this interrogative sentence can be represented as in (8):

(8) 〈…, C〉 + REQUEST_S1,S2_ (ASSERT(ψ)),

where C is conceived as a commitment space composed by a set of commitment states at the time the speaker S1 addresses the question to the addressee S2. With respect to this commitment space C, S1 requests to S2 whether the assumption is true that someone has not come. One of the most common rejecting answers to negative propositions found in Catalan was the isolated particle *sí* “yes” with a marked contradictory intonation contour, analyzed as in (9).

(9) S2 to S1: *Sí*_L+H^*^L!H%_ “Yes” [Yes, he/she HAS]
     [_ForceP_REJECT_S2,S1_ L+H^*^ L!H% [_ForceP_ ASSERT_S2,S1_ sí [_TP_ ha vingut]]]

By answering *sí* the speaker is asserting a specific proposition, which in order not to be in contradiction with the lexical contents of the particle must be a positive proposition such as φ, which corresponds to the TP of the discourse polar question. By means of the marked intonation pattern L+H^*^ L!H% the speaker S2 is expressing REJECT against the negative assumption of the negative question. (S) he is consequently asserting a commitment to the truth of the proposition corresponding to TP, that is φ, and at the same time rejecting ψ. The next interpretation move is that S2 expects that S1 will incorporate φ to the common ground. Therefore, the meaning of (9) can be represented as follows:

(10) 〈…, C〉 + REJECT_S2,S1_ + ASSERT [S2: φ] + [φ ∈ CG]

A speech act dynamic analysis of the most relevant rejecting strategies found in the Catalan answering system is summarized in Table 2. The contradicting answer *sí* “yes” (with L+H^*^ L!H%) to a negative question can be semantically analyzed as being composed of a REJECT operator (encoded by prosody and gesture) followed by ASSERT (encoded through the positive particle, followed by φ, which picks up the propositional discourse referent). The same semantic analysis can be applied to the other (most common) rejecting responses we obtained, namely a repetition of *sí* “yes” alone, *sí* “yes” followed by an explanation, *sí que* “yes that” + positive sentence, and *no, sí que* “no yes that” + positive sentence. In other words, when the intonation is unmarked, other strategies are used to express reject.

**Table 2:** **Speech act analysis of rejecting strategies in Catalan**.

**Rejecting strategies (Catalan)**	**Reject_S2,S1_**	**Assert_S2,S1_**	**φ**
*Sí*_L+H^*^L!*H*%_	L+H^*^ L!H%	*sí* “yes”	∅
*Sí, sí* “yes, yes”	∅	*sí sí* “yes yes”	∅
*Sí, sí* “yes, yes”	*sí* “yes”	*sí* “yes”	∅
*Sí* + explanation	∅	*sí* “yes”	Explanation
*Sí que* + positive sentence	∅	*sí que* “yes that”	Positive sentence
*No, sí que* +positive sentence	*no* “no”	*sí que* “yes that”	Positive sentence
Any of the above + rejection gestures	Rejection gestures (strong/repeated head nod, tilt, strong/slight eyebrow raising, shrug)	*sí* “yes”	Any of the above

Summing up, in Catalan the particle *sí* “yes” is the expression of the operator ASSERT, but it may also be the expression of REJECT only when it combines with a second *sí*^8^. Native speakers react against a negative assertion or a negative assumption of a negative polar question by means of a marked intonation, the negative particle *no*, or a marked gesture (strong/repeated head nod, tilt, strong/slight eyebrow raising, shrug). Sometimes the speech act of REJECT is not overtly expressed, but the interlocutor infers the expression of REJECT from context or from the contents of the explanation / positive sentence that follows the particle *sí*^9^.

#### Russian

Let us now turn to Russian. This language differs from a clearly polarity-based language such as Catalan in that, according to the results obtained, speakers reject a negative assertion or a negative presupposition associated with a negative polar question by means of a verb that is the echoic expression occurring in the discourse context, or by means of the negative particle *net* “no” followed by an echoic verb or an explanation, by a verb + explanation, by repeating *net* + explanation, by repeating the verb, or by asserting a positive sentence.

Suppose that S1 addresses the following request to S2:

(11) S1 to S2: *On ne prikhodil?*                  “Has he not come?”
       [_ForceP_REQUEST_S1,S2_   [_ForceP_ ASSERT_S1,S2_   [_NegP_ on ne prikhodil]]]

A rejecting answer to this negative question received two significant replies in our data, which are analyzed in (12) and (13).

(12) S2 to S1: *Prikhodil*                        has come [Yes, he did]
       [_ForceP_REJECT_S2,S1_ [_ForceP_ ASSERT_S2,S1_ [_TP_ prikhodil]]]
(13) S2 to S1: *Net, prikhodil*                 no has come
                                                            [Yes, he did]
       [_ForceP_REJECT_S2,S1_ net [_ForceP_ ASSERT_S2,S1_ [_TP_ prikhodil]]]

Notice that in (13) *net* corresponds to a REJECT particle^10^. Russian does not make use of any explicit marked intonation, although speakers tend to favor more HL^*^ L% than L^*^ L% for the expression of reject (see Figure 6). In this language, in addition to contextual information and the contents of the explanation/positive sentence that may follow the linguistic expression, some gestural patterns (strong/slight/repeated head nod, tilt/shake, strong/slight eyebrow rising, shrug) are relevant too at the time of identifying the expression of REJECT at the grammar-cognition interface. In those situations where S2 repeats the particle, the structure can be analyzed with two *net* particles under REJECT^11^. This analysis makes explicit an interesting contrast with a polarity-oriented language such as Catalan: Russian shows the repetition of the particle *net* “no” for REJECT, whereas Catalan shows the repetition of the particle *sí* “yes.”

(14) [_ForceP_REJECT_S2,S1_  net  net   [_ForceP_   ASSERT_S2,S1_   [_TP_ priejal]]]

The set of strategies used by our Russian informants are summarized in Table 3.

**Table 3:** **Speech act analysis of rejecting strategies in Russian**.

**Rejecting strategies (Russian)**	**Reject_S2,S1_**	**Assert_S2,S1_**	**φ**
V	∅	∅	V
*Net +* V/explanation	*net* “no”	∅	V/explanation
*Net, net* + explanation	*net, net* “no, no”	∅	Explanation
*Net, net* + explanation	*net* “no”	*net* “no”	Explanation
*V* + explanation	∅	∅	V + explanation/positive sentence
Any of the above + rejection gestures	Rejection gestures (strong/repeated/slight head nod, tilt/shake, strong/slight eyebrow raising, shrug)	Any of the above	Any of the above

As explained above in relation to the Catalan data, the next interpretation move is that S2 expects that S1 will incorporate φ to the common ground. Therefore, the meaning of (12), (13), and (14) can be represented as follows:

(15) 〈…, C〉 + REJECT_S2,S1_ + ASSERT [S2: φ] + [φ ∈ CG]

To summarize, in the last two sections we have shown that Catalan and Russian show asymmetries concerning the lexico-syntactic and prosodic strategies that speakers use in natural acts of production. Still, strong similarities have been shown concerning the gestural patterns used by native speakers of these languages. The analysis we have provided to account for the data obtained in our experimental work highlights the correspondence between these strategies and various speech act operators, namely REJECT and ASSERT.

## Conclusions

In this paper we have shown the results of a production experiment run with native speakers of Catalan and Russian at the time of confirming or rejecting previous input commitments: (positive or negative) assertions and (positive or negative) polar questions. We have analyzed the lexico-syntactic, prosodic and gestural strategies used in speech acts of CONFIRM and REJECT.

This work has shown that speakers of the two languages under study do not make use of exactly the same grammatical strategies at the time of expressing CONFIRM and REJECT to input commitments. In particular, the results lead to the conclusion that speakers of Catalan, a language that has been described as being polarity-based, can make use of lexico-syntactic strategies characteristic of truth-based systems at the time of expressing REJECT (e.g., the grammatical construction *no, sí que…* “no, yes that…”). Similarly, Russian, a language with a mixed system, shares with Catalan gestural strategies in the expression of both CONFIRM and REJECT. Therefore, the classification between polarity-based and truth-based languages has to be further refined.

Further, we have argued for the potential existence of a universal semantico-pragmatic strategy for rejecting negative propositions in speech act conversations, by which rejecting answers are optimally composed of a REJECT speech act operator applying over an ASSERT speech act operator. These operators may have a null morphophonological realization or be instantiated by a set of lexico-syntactic, prosodic, and gestural features. At this juncture, our general claim is that prosodic patterns and speech-accompanying gestures can crucially contribute “multidimensional meanings” which interact with other meanings contributed by lexico-syntactic features. Along with Ebert et al. (2011), we foresee that multidimensional semantic models are especially promising in the study of language production and understanding, and we advocate for the full integration of the pragmatic and semantic meanings contributed by prosody and gesture in linguistic research.

### Conflict of interest statement

The authors declare that the research was conducted in the absence of any commercial or financial relationships that could be construed as a potential conflict of interest.

## Footnotes

^1^This article will deal with answers to negative questions which encode *inner* negation, e.g., a bias toward a negative answer, as shown by the negative polarity item *either* (ia). In English, as in other languages, negative questions can also encode a bias toward a positive answer [e.g., the so-called *outer negation polar questions*, as shown by the positive polarity item *too* (ib)]. See Ladd (1981) and Romero and Han (2004) for this distinction. (i) a. Isn't Jane coming (either)?    b. Isn't Jane coming (too)?

^2^A 70% of Catalan daily use is quite typical for Catalan dominant speakers in the Barcelona area, which is a highly bilingual area where Catalan dominant speakers interact frequently with Spanish-dominant speakers.

^3^Table 1 only includes question prompts (e.g., *Has the deliveryman come?, Has the deliveryman not come yet?)*. However, as mentioned before, the corresponding assertion prompts were also used in each condition (e.g., *I suppose that the deliveryman has already come, I see that the delivery man hasn't come yet*).

^4^Unfortunately, this same linguistic test cannot be applied in the case of Russian, since the target negative polarity items *either* and *too* correspond to the same Russian word *tozhe*.

^5^The two only cases in which a negative presupposition was rejected by uttering a *no*-word followed by a positive sentence (see “No, sí que + positive sentence” in Figure 1) were produced together with a head tilt and a shoulder shrug.

^6^For alternative syntactic approaches based on purely syntactic ellipsis accounts, see among others: Holmberg (2001, 2003, 2013) and Kramer and Rawlins (2009). For a semantic perspective on the study of so-called polarity particles as mechanisms for confirming and reversing reactions, see Farkas and Bruce (2010) and Farkas and Roelofsen (2012). Although we acknowledge the series of pragmatic studies on negative questions and their potential answers developed both at a theoretical level (within a dynamic semantic theory of the rhetorical connections between utterances in a discourse or dialogue; see Reese, 2006) and at an experimental level (Noveck, 2009; Levinson, 2010), we advocate for an analysis in terms of speech acts and changing commitment states in conversational moves (Cohen and Krifka, 2011; Krifka, 2013, in press).

^7^See also Krifka (2013, in press) for an analysis of response particles in terms of propositional anaphors, and for an analysis of assertions and reactions to assertions within a dynamic semantic theory of speech acts.

^8^Notice that the sequence *s*í, *s*í has two possible analyses, depending on whether the two *s*í are the instantiation of ASSERT (as expected from a basic polarity-based language) or one of them instantiates REJECT. We have no conclusive arguments for either one or the other.

^9^See Farkas and Bruce (2010) and Krifka (2013) and Roelofsen and Farkas (To appear) for a discussion of other lexical strategies found in polarity-based languages: the particles *si* in French and *doch* in German, which lexicalize both REJECT and ASSERT, and the particle *ba* in Romanian, which lexicalizes only REJECT (and as such can be followed by the polarity ASSERT particles *da* and *nu*). In Russian, a language that shows strategies of mixed answering systems, *da net* is also a possible realization of both REJECT and ASSERT.

^10^This example should be distinguished from (3,A1) above, where *net* “no” is the instantiation of ASSERT, followed by a negative sentence *ne vernu* “I will not return [the book].” In this sense, the particle *net* can be both the expressions of ASSERT and REJECT.

^11^An alternative analysis would postulate one *net* under REJECT and a second *net* under ASSERT. In support of this alternative analysis we would like to point out that, in contrast to particles *yes*/no in English but close to particles *ja*/nein in German and *s*í/no in Catalan, Russian *da*/net may occur next to a conditional adverb or in complement position to a *believe* verb.

**Table:**

(i) a. Did he steal a book?	–If ?‘??yes, he must give it back. (English)
	–John believes ?‘??yes.
b. *On ukral knigu?*	–*Jesli da, on dolzen ee vernut'* (Russian)
he stole book.ACC	if yes, he must it.ACC give-back
	–*Petr dumajet, cto da*.
	Peter thinks that yes
