# Supplementary material for: Is there a universal answering strategy for rejecting negative propositions? Typological evidence on the use of prosody and gesture
Source: Front Psychol. 2015 Jul 7;6:899. doi: 10.3389/fpsyg.2015.00899 (PMC4493367; doi:10.3389/fpsyg.2015.00899)
Supplement: Supplementary file 1 [file DataSheet1.DOCX]

**APPENDIX**

**English translation of one of the discourse contexts presented to participants.**

**Situation**

Some time ago, your flatmate and you subscribed to a newspaper which is delivered every Saturday afternoon. Today is Saturday, but your flatmate will not be at home, so it is you who is responsible for opening the door to the deliveryman.

**Linguistic prompts**

**Positive Question – Confirming Answer**

When your flatmate arrived at night, she asks you:

“Has the deliveryman come?”

You confirm that he has come. What would you say?

**Positive Assertion – Confirming Answer**

When your flatmate arrives at night, she says to you:

“I suppose that the deliveryman has already come”

You confirm that he has come. What would you say?

**Positive Question – Rejecting Answer**

When your flatmate arrives at night, the delivery man has not come yet. She asks you:

“Has the deliveryman come?”

You deny that he has come. What would you say?

**Positive Assertion – Rejecting Answer**

When your flatmate arrives at night, the delivery man has not come yet. She says to you:

“I suppose that the deliveryman has already come”

You deny that he has come. What would you say?

**Negative Question – Confirming Answer**

When your flatmate arrives at night, she does not see the newspaper in the kitchen, where you usually leave it, and then she asks you:

“Has the deliveryman not come yet?”

You confirm that he has not come. What would you say?

**Negative Assertion – Confirming Answer**

When your flatmate arrives at night, she does not see the newspaper in the kitchen, where you usually leave it, and then she says to you:

“I see that the deliveryman has not come yet”

You confirm that he has not come. What would you say?

**Negative Question – Rejecting Answer**

When your flatmate arrives at night, she does not see the newspaper in the kitchen, where you usually leave it. Actually, the deliveryman has come, but you took the newspaper to your room and you forgot to return it to the kitchen. As she does not see the newspaper in the kitchen, she asks you:

“Has the deliveryman not come yet?”

You contradict her. What would you say?

**Negative Assertion – Rejecting Answer**

When your flatmate arrives at night, she does not see the newspaper in the kitchen, where you usually leave it. Actually, the deliveryman has come, but you took the newspaper to your room and you forgot to return it to the kitchen. As she does not see the newspaper in the kitchen, she says to you:

“I see that the deliveryman has not come yet”

You contradict her. What would you say?
